# Enterotoxin-producing *Escherichia coli* O169:H41, United States

**DOI:** 10.3201/eid1003.030268

**Published:** 2004-03

**Authors:** Mark E. Beatty, Cheryl A. Bopp, Joy G. Wells, Kathy D. Greene, Nancy D. Puhr, Eric D. Mintz

**Affiliations:** * Centers for Disease Control and Prevention, Atlanta, Georgia USA 30333

**Keywords:** *Escherichia coli*, enterotoxins, gastrointestinal diseases, disease outbreaks, drug resistance

## Abstract

From 1996 to 2003, 16 outbreaks of enterotoxigenic *Escherichia coli* (ETEC) infections in the United States and on cruise ships were confirmed. *E. coli* serotype O169:H41 was identified in 10 outbreaks and was the only serotype in 6. This serotype was identified in 1 of 21 confirmed ETEC outbreaks before 1996.

Enterotoxigenic *Escherichia coli* (ETEC) is an important cause of diarrhea in the developing world and among travelers and is increasingly recognized as a cause of outbreaks in the United States (*1*). Dalton et al. reviewed confirmed ETEC outbreaks that occurred both in the United States and among passengers on cruise ships that docked in U.S. ports from 1975 through 1995 (*2*). Twenty-one such outbreaks caused by 17 different ETEC serotypes occurred during this period; 7 (33%) occurred among cruise ship passengers (*2*). Because laboratory tests for the identification of ETEC are not widely available, outbreaks caused by ETEC may escape recognition, and healthcare workers may miss opportunities for treatment and prevention. To improve recognition of ETEC outbreaks, Dalton proposed that specimens from outbreaks of gastroenteritis that meet certain criteria be referred for ETEC testing at a public health reference laboratory. These outbreaks include those for which routine stool cultures have not yielded an etiologic agent and those which are characterized by an incubation period of 24 to 48 hours, a duration of illness >60 hours, and a diarrhea-to-vomiting prevalence ratio of >2.5 (*2*).

## The Study

For the 8-year period 1996 through 2003, we reviewed all suspected ETEC outbreaks solely or jointly investigated by the Foodborne and Diarrheal Diseases Branch at the Centers for Disease Control and Prevention (CDC). In accordance with Dalton et al., we defined a confirmed ETEC outbreak as one in which ETEC isolates of the same serotype were isolated from >3 ill persons and no other viral or bacterial pathogens were identified, or one in which ETEC isolates of the same serotype were isolated from >10 ill persons and no more than one other bacterial or viral pathogen was identified in a single stool specimen. Stool specimens collected during these investigations were routinely cultured for *Salmonella*, *Shigella*, *Campylobacter*, *E. coli* O157:H7, *Yersinia*, and *Vibrio* spp. In many instances, these specimens were also tested for Noroviruses**.**

To identify ETEC, patient specimens were plated to MacConkey agar, and individual colonies or sweeps of confluent growth were tested by polymerase chain reaction (PCR) for heat-labile (LT) and heat-stable (ST) enterotoxin genes (*3*). Using standard methods, we serotyped LT- or ST-positive isolates for O and H antigens. ETEC isolates were tested by the disk-diffusion method for susceptibility to ampicillin, amoxicillin/clavulanic acid, ceftriaxone, chloramphenicol, ciprofloxacin, gentamicin, kanamycin, nalidixic acid, streptomycin, sulfisoxazole, tetracycline, and trimethoprim-sulfamethoxazole (*4*). A PCR–restriction fragment length polymorphism test was used to identify the H41 gene in selected nonmotile *E. coli* O169 isolates (*5*).

## Conclusions

During the 8-year study period, CDC received isolates from 59 outbreaks for ETEC testing. Sixteen met the criteria for our definition of a confirmed ETEC outbreak; three occurred on international cruise ships that docked in U.S. ports and 12 occurred in the United States (Table). We identified ETEC in specimens from six other outbreaks that did not meet these criteria, either because ETEC isolates of the same serotype were isolated from only two persons (n = 2) or because we isolated additional bacterial pathogens from the specimens (n = 4).

**Table:** Characteristics of enterotoxigenic *Escherichia coli* (ETEC) outbreaks,^a^ United States, 1996–2003

No.	Mo/y	Location	Setting (reference)	Presumed source	No ill	% Diarrhea (bloody)	% Vomiting	Median incubation, h (range)	Median illness duration, days (range)	No isolates	Serotype/toxin type^b^	Antimicrobial resistance^c^	
1	3/96	Caribbean	Cruise ship	Drinking water	652	98 (9)	19	–	–	6	O169:H41/ST	Tc	
										1	O6:H16/ LT,ST	Sensitive	
										1	O27:H7/ST	St, Su, Tc	
										1	O34:H10/ST	Tc	
2	4/97	Caribbean	Cruise ship (5)	Drinking water, ice	429	100 (6)	14	–	>3	8	O169:H41/ST	Tc	
										3	O148:H28/ LT,ST	Tc	
										1	O78:H12/ST	Ch, St, Su, Tc, TmS	
										1	O27:H7/ST	St, Su, Tc	
3	4/97	California	Restaurant	Beans, enchilada, tacos, rice, tortilla chips	41	95	17	24 (5–85)	2 (0.3–4)	11	O27:H7/ST	Sensitive	
4	6/97	Massachusetts	Boxed lunch	Tomato, mozzarella salad	33	97	33	48 (24–96)	3.7 (1-7)	5	O25:NM/ST	Sensitive	
5	7/97	Minnesota	Catered party	Fresh vegetables	15	100	13	37 (8-158)	3.2 (0.4–6)	4	O169:H41/ST	Tc	
6	4/98	Mexico-Hawaii	Cruise ship	–	397	96	33	–	–	3	O6:H16/ LT,ST	Sensitive	
										1	O6:H16/ LT,ST	St, Tc	
										2	O169:H41/ST	Tc	
										1	O148:H28/ LT,ST	St, Su, Tc	
										1	O148:H28/ LT,ST	Tc	
										1	O27:H7/ST	St, Su, Tc	
7	6/98	Illinois	Catered parties	Potato, macaroni, egg salads	916	100	8	50 (40–76)	5 (2–9)	11	O6:H16/ LT,ST	Sensitive	
8	8/98	Minnesota	Restaurant (6)	Parsley	66	100 (0)	6	25	8	7	O6:H16/ LT,ST	Ap, St, Su, Tc	
										1	O159:H4/LT	Ap, St, Su, Tc, TmS	
										1	O27:H7/ST	St, Su, Tc	
9	9/98	Minnesota	Restaurant	–	5	100 (0)	0	48	6	3	O169:H41/ST	St, Su, Tc	
10	5/00	Washington	Cruise ship	Basil	100	100	5	40 (27–67)	10 (0.5–21)	3	O169:H41/ST	Tc	
11	6/00	New York	Banquet	–	40	97 (0)	3	48 (24–96)	3 (1–8)	5	O169:H41/ST	Tc	
12	7/00	Utah	Wedding rehearsal	–	45	100	29	33 (18–59)	2.3 (1–3)	5	O27:H7/ST	St, Su, Tc	
13	7/01	Wisconsin	Catered party	Quesadillas, fajitas, nacho chips, beans	21	100	–	38 (9–70)	6 (1–8)	3	O169:H41/ST	Tc	
14	8/01	Illinois	Catered party	–	24	100	0	–	–	3	O169:H41/ST	Ap, Tc	
										1	O169:H41/ST	Tc	
										2	O6:H16/LT,ST	Sensitive	
										1	O25:NM/ST	Ap	
15	10/02	Oregon	Catered party	Garlic chicken lasagna	40	98 (13)	15	72 (24–144)	5	3	O27:H7/ST	St, Su,Tc	
16	8/03	Tennessee	Catered party	Catfish, coleslaw	41	81 (0)	5	2	2.5	12	0169:H49	Tc	

^a^An outbreak is defined as ≥3 ill persons infected with the same ETEC serotype and no other viral or bacterial pathogens, or >10 ill persons with the same serotype and no more than one other bacterial or viral pathogen identified. ^b^NM, nonmotile; LT, heat-labile toxin; ST, heat-stable toxin. ^c^Ap, ampicillin; Amc, amoxicillin/clavulanic acid; Ch, chloramphenicol; St, streptomycin; Su, sulfisoxazole; Tc, tetracycline; TmS, trimethoprim-sulfamethoxazole.

The 16 ETEC outbreaks had a median of 41 ill persons per outbreak (range 5−916), for a total of 2,865 patients. From 81% to 100% of ill persons in each outbreak reported diarrhea; less frequently reported symptoms included abdominal cramps (66%–90%), fever (0%–73%), nausea (44%–70%), and vomiting (0%–33%). In the 15 outbreaks in which diarrhea and vomiting were reported, the median “diarrhea-to-vomiting prevalence ratio” (the percentage of patients who reported diarrhea divided by the percentage of patients who reported vomiting) was 7.7 (range 2.9–undefined: The upper limit of the range is undefined because in two outbreaks all ill persons interviewed denied vomiting). In nine outbreaks with sufficient data, incubation periods among individual patients were 5–158 hours. The median incubation period was 24–48 hours for 10 of the 12 outbreaks for which it could be calculated. The duration of illness, reported in 11 outbreaks, was 0.3–21 days. The median duration of illness was >60 hours in 11 of the 13 outbreaks for which it could be calculated.

A vehicle was implicated in 11 (69%) outbreaks. Unbottled ship’s water or beverages containing ice prepared on board the ship were implicated in the two outbreaks on cruise ships that had docked in foreign ports. Although no problems with chlorination of bunkered water were documented in these two outbreaks, this problem has been seen in previous waterborne ETEC outbreaks aboard cruise ships (*6*). Basil served on board ship was implicated as the source of ETEC in the remaining cruise ship outbreak (on a ship that docked only in U.S. ports). One other outbreak was attributed to a fresh herb (parsley) served raw (*7*). Salads made with raw vegetables were implicated in four other domestic outbreaks.

If we define a strain as each ETEC serotype identified during an outbreak that has a unique antimicrobial resistance pattern, we identified a total of 30 strains representing eight different serotypes in specimens from the 16 outbreaks (Table). In five outbreaks, we isolated more than one ETEC serotype. Heat-stable toxin (ST)-producing *E. coli* O169:H41 was the most commonly identified serotype. This serotype was identified as the only pathogen in specimens from six outbreaks in the United States and was identified along with other ETEC serotypes in four additional outbreaks. Three of these four outbreaks occurred on cruise ships. In 21 previously reported ETEC outbreaks, *E. coli* O169:H41 had been isolated only once, from an outbreak that occurred among international cruise ship passengers in 1995, in which another ETEC serotype predominated (*2*). By all of the basic epidemiologic and clinical characteristics that we analyzed, outbreaks in which *E. coli* O169:H41 was identified alone, or in combination with other serotypes, did not appear to differ from outbreaks in which this emerging strain was not identified.

Resistance to antimicrobial agents remained common among ETEC isolates (Table). Twenty-four (80%) of the 30 strains were resistant to tetracycline, 11 (38%) were resistant to sulfisoxazole, 4 (13%) were resistant to ampicillin, and 2 (7%) were resistant to trimethoprim-sulfamethoxazole. All strains of O169:H41 were resistant to tetracycline, and two were also resistant to at least one additional antimicrobial drug. Only two outbreaks were caused exclusively by pan-sensitive ETEC strains (7%).

A comparison of ETEC outbreaks reported to CDC from 1996 through 2003 with those from previous years shows that outbreaks on cruise ships and in the United States continue to occur and that antimicrobial resistance among ETEC isolates remains common. Raw vegetables and herbs have been increasingly implicated as the vehicles for ETEC outbreaks in recent years. This finding is in keeping with an increase in produce-associated outbreaks among other foodborne bacterial pathogens (*8*). Finally, a new ETEC serotype, ST-producing O169:H41, has become predominant.

The first report of O169:H41 was in association with a foodborne outbreak in 1991 in Japan, where this serotype continues to be isolated (*9**–**11*). Hamada reported four outbreaks that occurred from June 1997 to August 1998; the largest of these outbreaks had a 57% attack rate and resulted in approximately 2,800 cases. All four outbreaks occurred at either restaurants or catered events (*11*). Contaminated wakame seaweed was implicated in one of the outbreaks and considered the likely cause in another (*11*). Nishakawa et al. report that O169:H41 has become the most prevalent ETEC serotype in Japan (*12*).

In 1995, CDC detected this serotype for the first time during an outbreak on a Caribbean cruise ship. It was identified during two additional Caribbean cruise ship outbreaks before causing a domestic outbreak in Minnesota in 1997. In addition to being identified in 10 of the 16 ETEC outbreaks that met our criteria, O169:H41 was also a predominant serotype in 4 of the 6 ETEC outbreaks that did not meet our criteria for a confirmed outbreak, either because the serotype was isolated from two persons only or because an additional pathogen was also isolated in the outbreak.

The emergence and eventual predominance of O169:H41 in the United States and Japan may have important implications for ETEC vaccine producers. Nishikawa et al. characterized strains of O169:H41 from Japan and reported that they are not clonal and that they possess a novel colony-forming factor (*12*).

From 1996 through 1999, laboratory-confirmed ETEC outbreaks represented 0.2% of all foodborne outbreaks reported to CDC (*13*). This number is likely to be an underestimate because special diagnostic tests are required to confirm ETEC. Seventy-one percent of outbreaks of foodborne illness reported to CDC during this period were of unknown cause. In a previous study, Hall et al. demonstrated that the epidemiologic and clinical syndrome in 1.1% of outbreaks of unknown cause reported to CDC from 1982 through 1989 was compatible with infections caused by ETEC or STEC (*14*). ETEC is also responsible for some episodes of sporadic diarrheal disease. When researchers systematically looked for it, they isolated ETEC from 1.4% of stool samples from patients visiting urban and rural health maintenance organization clinics in Minnesota for diarrhea (*15*). Our data suggest that the clinical criteria proposed by Dalton et al. for suspecting ETEC as a cause of an outbreak of unknown cause (median incubation 24–48 hours, mean or median duration >60 hours, and diarrhea-to-vomiting prevalence ratio >2.5) remain valid.

The epidemiology of ETEC outbreaks in the United States is changing, but the incidence of these outbreaks does not appear to be decreasing. Researchers cannot use routine stool cultures to detect ETEC, and delays in stool sample collection for >7 days greatly reduces yield (*16*,*17*). For outbreaks that meet the clinical profile and for which routine stool diagnostic tests have not yielded an enteric pathogen, physicians and public health authorities should send *E. coli* isolates to reference laboratories, such as CDC, for ETEC testing.
