# Correction, Vol. 10, No. 3

**DOI:** 10.3201/eid1004.C11004

**Published:** 2004-04

On p. 519, in the table entitled “Characteristics of enterotoxigenic *Escherichia coli* (ETEC) outbreaks, United States, 1996–2003,” the serotype of the strain associated with outbreak number 16 was O169:H41 not O169:H49.

